# Hand-Book of Chemistry, Theoretical, Practical, and Technical

**Published:** 1854-08

**Authors:** 


*Hand-Book of Chemistry, Theoretical, Practical, and Technical.* By F. A. ABEL, Professor of Chemistry at the Royal Military Academy, Woolwich, and Assistant Teacher of Chemistry at St. Bartholomew's Hospital; and C. L. BLOXAM, formerly First Assistant to the Roy l College of Chemistry. With a Preface, by Dr. HOFFMAN; and numerous Illustrations on Wood.—Philadelphia: Blanchard and Lea. 1854.

THIS is a volume of 680 pages; embracing, besides an elementary treatise on chemistry, and an account of the various elementary bodies, detailed instruction in the various modes of analysis and other chemical manipulations. To the student who wishes to make himself familiar with all the details of practical chemistry, we should think the work one of great value. Its contents, however, do not admit of a brief analysis; and we must, therefore, content ourselves with this simple statement in reference to their nature and value. D.

